# Haplotype Motif-Based Models for KIR-Genotype Informed Selection of Hematopoietic Cell Donors Fail to Predict Outcome of Patients With Myelodysplastic Syndromes or Secondary Acute Myeloid Leukemia

**DOI:** 10.3389/fimmu.2020.584520

**Published:** 2021-01-19

**Authors:** Johannes Schetelig, Henning Baldauf, Linda Koster, Michelle Kuxhausen, Falk Heidenreich, Liesbeth C. de Wreede, Stephen Spellman, Michel van Gelder, Benedetto Bruno, Francesco Onida, Vinzenz Lange, Carolin Massalski, Victoria Potter, Per Ljungman, Nicolaas Schaap, Patrick Hayden, Stephanie J. Lee, Nicolaus Kröger, Kathy Hsu, Alexander H. Schmidt, Ibrahim Yakoub-Agha, Marie Robin

**Affiliations:** ^1^ Medizinische Klinik und Poliklinik I, University Hospital Dresden, Dresden, Germany; ^2^ DKMS Clinical Trials Unit, Dresden, Germany; ^3^ EBMT Data Office Leiden, Leiden, Netherlands; ^4^ Center for International Blood and Marrow Transplant Research, Minneapolis, MN, United States; ^5^ Leiden University Medical Center, Department of Biomedical Data Sciences, Leiden, Netherlands; ^6^ Maastricht University Medical Center, Department of Internal Medicine, Maastricht, Netherlands; ^7^ A.O.U. Citta della Salute e della Scienza di Torino, Turin, Italy; ^8^ Fondazione IRCCS Ca’Granda Ospedale Maggiore Policlinico, University of Milan, Milan, Italy; ^9^ DKMS Life Science Lab, Dresden, Germany; ^10^ GKT School of Medicine, London, United Kingdom; ^11^ Karolinska University Hospital and Karolinska Institutet, Stockholm, Sweden; ^12^ Radboud University Medical Centre, Nijmegen, Netherlands; ^13^ St. James’s Hospital, Dublin, Ireland; ^14^ Fred Hutchinson Cancer Research Center, Seattle, WA, United States; ^15^ University Hospital Eppendorf, Hamburg, Germany; ^16^ Memorial Sloan Kettering Cancer Center, New York & Scientific Director, CIBMTR Immunobiology Working Committee, New York City, NY, United States; ^17^ CHU de Lille, LIRIC, INSERM U995, Université de Lille, Lille, France; ^18^ Hopital Saint-Louis, APHP, Université de Paris, Paris, France

**Keywords:** KIR, KIR2DS1, KIR3DL1, hematopoietic stem cell transplantation, donor selection, unrelated donor

## Abstract

Results from registry studies suggest that harnessing Natural Killer (NK) cell reactivity mediated through Killer cell Immunoglobulin-like Receptors (KIR) could reduce the risk of relapse after allogeneic Hematopoietic Cell Transplantation (HCT). Several competing models have been developed to classify donors as KIR-advantageous or disadvantageous. Basically, these models differ by grouping donors based on distinct KIR–KIR–ligand combinations or by haplotype motif assignment. This study aimed to validate different models for unrelated donor selection for patients with Myelodysplatic Syndromes (MDS) or secondary Acute Myeloid Leukemia (sAML). In a joint retrospective study of the European Society for Blood and Marrow Transplantation (EBMT) and the Center for International Blood and Marrow Transplant Research (CIBMTR) registry data from 1704 patients with secondary AML or MDS were analysed. The cohort consisted mainly of older patients (median age 61 years) with high risk disease who had received chemotherapy-based reduced intensity conditioning and anti-thymocyte globulin prior to allogeneic HCT from well-matched unrelated stem cell donors. The impact of the predictors on Overall Survival (OS) and relapse incidence was tested in Cox regression models adjusted for patient age, a modified disease risk index, performance status, donor age, HLA-match, sex-match, CMV-match, conditioning intensity, type of T-cell depletion and graft type. *KIR* genes were typed using high-resolution amplicon-based next generation sequencing. In univariable and multivariable analyses none of the models predicted OS and the risk of relapse consistently. Our results do not support the hypothesis that optimizing NK-mediated alloreactivity is possible by KIR-genotype informed selection of HLA-matched unrelated donors. However, in the context of allogeneic transplantation, NK-cell biology is complex and only partly understood. KIR-genes are highly diverse and current assignment of haplotype motifs based on the presence or absence of selected KIR genes is over-simplistic. As a consequence, further research is highly warranted and should integrate cutting edge knowledge on KIR genetics, and NK-cell biology into future studies focused on homogeneous groups of patients and treatment modalities.

## Introduction

To harness natural killer (NK) cells for graft-versus-leukemia reactions by selecting donors based on KIR genotype information could further improve outcome after allogeneic hematopoietic cell transplantation (alloHCT). Evidence for the potential of allogeneic NK cells to attack cancer cells comes from a series of clinical studies demonstrating activity of haploidentical NK cell infusion or transplantation for patients with relapsed or refractory acute myeloid leukemia (AML) or myelodysplastic syndrome (MDS) (1–5). NK cytotoxicity may be triggered by activating Killer Immunoglobulin like Receptors (KIRs), which encounter their cognate ligand on target cells, or by inhibitory KIRs, which do not encounter their cognate ligands on target cells. KIR–KIR–ligand interactions may elicit NK-alloreactivity also in the setting of HLA-compatible related or unrelated donor transplantation. This was supported by a series of retrospective registry studies which reported associations between certain donor-patient KIR genotype patterns and the risk of relapse after alloHCT (6–13).

The human *KIR* region has a complex architecture and comprises 15 genes and 2 pseudogenes. Six *KIR* genes (*KIR2DL1*, *KIR2DL2*/*3*, *KIR2DL5*, *KIR3DL1*, *KIR3DL2*, *KIR3DL3*) encode inhibitory receptors with long (L) cytoplasmic tails, while six genes encode receptors with short (S) activating cytoplasmic tails (*KIR2DS1*, *KIR2DS2*, *KIR2DS3*, *KIR2DS4*, *KIR2DS5*, *KIR3DS1*). Two pseudogenes are not expressed as proteins (*KIR2DP1*, *KIR3DP1*). Four genes are present in most of the common haplotypes and have been designated as “framework” genes (*KIR3DL3*, *KIR3DP1*, *KIR2DL4*, *KIR3DL2*) *(* 14, 15). *KIR* genes exhibit substantial allelic diversity (16). Furthermore, *KIR* haplotypes vary with respect to the presence or absence of specific *KIR* genes and are subject to copy number variation (17, 18).

Additionally, when improved sequencing technology allowed for allele-level resolution of *KIR* genes it became clear that absence/presence typing was not sufficient to determine the functional status of KIRs (18, 19). For example, the third most common allele of *KIR3DL1*, KIR3DL1*004, accounts for 17% of all *KIR3DL1* genes but is not expressed on the cell surface. *KIR3DL1* allotypes differ with respect to their expression patterns (20). In the context of HIV infection, certain subtype combinations of *KIR3DL1* allotypes together with their cognate ligand Bw4 were strongly associated with the risk of progression to AIDS (21). Genetic information on presence versus absence alone might thus lead to wrong conclusions about function. However, individual *KIR* genes show extensive sequence polymorphism with 1110 alleles currently named in total (IPD-KIR Database, Release 2.9.0 as of July 2020) (22). Allelic diversity across genes ranges from 16 alleles for *KIR2DS3* and *KIR2DS1* to 183 alleles for *KIR3DL1*. As a consequence, the development of cutting edge typing technology that could generate allelic and copy number results was critical to move the research field forward (16).

Against this background we set out to validate an HLA-matched unrelated donor selection algorithm for patients with AML and MDS which was essentially aimed at reducing the inhibitory potential of donor NK cells and increasing the activating potential by donor selection based on information on *KIR2DS1* and *KIR3DL1* (12, 13). We recently reported results of a study in AML patients where we failed to replicate the findings of the original report (23). Here, we present the data on patients with MDS or secondary AML. Again, we were unable to confirm the *KIR3DL1*/*KIR2DS1*- based donor selection algorithm. In addition, we tested other major models to predict the risk of relapse and death based on donor *KIR* genotype information.

## Methods

### Inclusion Criteria

We conducted a joint study of the European Society for Blood and Marrow Transplantation (EBMT) and the Center for International Blood and Marrow Transplant Research (CIBMTR) on the impact of KIR genotype information on patient outcome after alloHCT for MDS or secondary AML. This study used DNA samples from stem cell donors which were stored at the Collaborative Biobank (www.cobi-biobank.de). All stem cell donors had provided written informed consent when they contributed a sample to the biobank. The study was approved by the responsible Ethical Committee at the Technische Universtität Dresden, Germany. Access to medical data was approved by the Review Boards of the Chronic Malignancies Working Party of EBMT, the Immunobiology Working Committee of the CIBMTR and the National Marrow Donor Program Institutional Review Board.

Patient inclusion criteria were first allogeneic HCT from an unrelated donor between January 2008 and December 2017, a diagnosis of Myelodysplastic Syndrome (MDS) or secondary AML at HCT, and age above 18 years with an available donor sample in the Collaborative Biobank.

### Sample Identity

Donor information was mapped to the medical data of the patient using the Donor ID as a key. In order to rule out errors during the mapping process, all donor samples were typed for *HLA* and *KIR* genes. Information on the *HLA*-genotype was used to double-check sample identity by comparing the typing result with the original typing results for that donor and by checking HLA-compatibility with the corresponding patient information. The HLA compatibility between donors and recipients was assessed based on two-field information for *HLA-A, -B, -C, -DRB1* and -*DQB1*. Donor-recipient pairs, whose HLA-compatibility could not be confirmed, were excluded.

### KIR Genotyping

Genotyping was performed using a high-resolution short-amplicon-based next generation sequencing workflow. *KIR* typing at the allele-level was based on sequencing of exons 3, 4, 5, 7, 8, and 9 and subsequent bioinformatic analysis as described previously (16).

### Classification Models Into KIR Advantageous and Disadvantageous Donors

*HLA-C* alleles were grouped in C1 and C2 ligands and *HLA-B* alleles were grouped into Bw4-80I/Bw-80T/Bw6 epitope bearing ligands based on information retrieved from https://www.ebi.ac.uk/ipd/kir/ligand.html. Information on KIR3DL1 and KIR2DS1 and their cognate ligands was grouped according to publications by Venstrom et al. (12) and Boudreau et al. (13). Further, we classified donors according to A versus B haplotype motifs using definitions for haplotype assignment as provided by Cooley et al. (10, 11). Finally, we calculated scores for selected additive models which integrate information on KIR-ligand combinations of donor-recipient pairs. We calculated the functional inhibitory KIR count by assigning a score of 1 for donor *KIR2DL1*, *KIR2DL2*, *KIR2DL3*, and *KIR3DL1* when the cognate ligands were exhibited by patient HLA molecules as described in the original paper by Boelen et al. (24). As an extension of this count we also calculated the weighted inhibitory score using the published weights for functional KIR-ligand pairs as follows: Inhibitory score = (1 if functional KIR2DL1) + (1 if strong functional KIR2DL2 or 0.5 if weak functional KIR2DL2) + (0.75 if functional KIR2DL3) + (1 if functional KIR3DL1) (24).

The score developed by Krieger et al. integrates information on inhibitory and activating KIR-ligand interactions (25). Two versions exist, a non-weighted version which incorporates the inhibitory missing KIR-ligand Score (IM-KIR Score) with assigned scores per interaction, and a weighted version (w-KIR Score). Both versions were calculated according to the original publication (25).

### Information Used for Risk Adjustment

MDS and sAML were grouped by adopting definitions from the World Health Organization classification of myeloid neoplasms and acute leukemia (26).

Using information on the genetic risk and disease stage at transplantation from EBMT Minimal Essential Data Forms, we calculated a simplified Disease Risk Index (DRI) for MDS and sAML. For this purpose, cytogenetic risk was classified according to the rules for the refined DRI (27) except for chromosome 17p abnormalities which were assigned to the adverse risk group. For patients with missing stage, disease or cytogenetic risk information, DRI group was imputed based on largest frequencies reported in the publication of the refined DRI. The intensity of conditioning regimens was classified according to working definitions of EBMT and CIBMTR (28).

Risk adjustment in the context of multivariable regression models included information on patients’ performance status, age, sex, CMV serostatus, disease risk index, conditioning intensity, T-cell depletion, HLA-matcing, donor age, donor sex, and donor CMV serostatus.

### Primary Endpoint and Power Considerations

Event-free Survival (EFS) was selected as primary endpoint and death, relapse or progression (whichever occurred first) were defined as events for EFS. The study was designed to validate the effect of the classification of donor *KIR2DS1* and *KIR3DL1* information on predicting EFS as published by Boudreau et al. (13). In sample size estimations for the comparison of strongly inhibiting versus weakly inhibiting donor-patient *KIR3DL1 HLA-B* subtype combinations, we calculated that data from approximately 1,700 patients were required to detect a 17% reduction in EFS events with a two-sided type I error of 5% at a power of 80%. Since we failed to validate this model in patients with AML (23), we extended the scope of this study and also evaluated alternative models for donor *KIR* genotype classification. No formal adjustment of the type I error was made for multiple testing for these exploratory analyses. The *post hoc* power for each comparison was calculated with Schoenfeld’s formula based on the given number of events and the reported effect estimates, with a two-sided 5% type I error probability (29).

### Statistical Analysis

Relapse or progression was selected as the major secondary endpoint. Additional endpoints were Non-Relapse Mortality and Overall Survival. Death without previous relapse or progression was defined as non-relapse death. EFS and Overall Survival (OS) probabilities were calculated with the Kaplan-Meier estimator and between-group comparisons were performed with the log-rank test. Relapse/progression and Non-Relapse Mortality were considered as competing risks and univariable comparisons built on cumulative incidence curves. Univariable comparisons for these endpoints were performed with the Gray Test. All time-to-event endpoints were evaluated in (cause-specific) multivariable Cox proportional hazards regression models. Effect sizes were reported as hazard ratios together with 95%-confidence intervals. We performed subgroup analyses for the effect of *KIR* genotype-based classifications in subpopulations defined by variations in the transplant procedure which could have an impact on NK alloreactivity, e.g. myeloablative conditioning versus reduced-intensity conditioning/non-myeloablative conditioning, use of Anti-Thymocyte Globulin (ATG) and of total body irradiation.

The proportionality assumption was checked for each covariable for the main models analyzing Overall Survival and relapse by means of plots of scaled Schoenfeld residuals and the test of Grambsch and Therneau (30).

## Results

### Patient Characteristics

Mapping of patients and donors resulted in 1,836 donor-recipient pairs. Data from three donor-recipient pairs were excluded because sample identity could not be confirmed. Typing of 108 samples failed because the DNA quantity or quality was too low for the workflow. No data on outcomes was available for 21 patients. The final statistical analysis set thus contained information on 1,704 patients.

The median age at allogeneic HCT was 61 years (range from 18 to 83 years). Indication for allogeneic HCT was secondary AML for 28% of patients, MDS for 63% of patients and MDS/MPN overlap syndromes in 9% of patients. Disease risk was assessed as intermediate, high or very high in 41, 58, and 0.6%, respectively. Patient and donor pairs were 10/10 matched in 79% of pairs, whereas a one locus mismatch was reported for 20% of pairs. Myeloablative, reduced-intensity and non-myeloablative conditioning regimens were used in 31, 56, and 11% of patients, respectively. Anti-Thymocyte globulin was administered as GVHD-prophylaxis in 56% and Alemtuzumab in 9% of patients. Thirty five percent of patients received no T-cell depletion, and 1% received an *ex vivo* T-cell depleted graft. Peripheral Blood Stem Cells (PBSC) and Bone Marrow (BM) were used as graft source in 93 and 7% of patients, respectively. Further details and the distribution of patient characteristics are given in **Table 1**.

**Table 1:** Patient and treatment characteristics.

Parameter		Total Cohort	
		N	(%)
Patient Numbers		1704	(100)
Patient Sex	Male	1009	(59)
	Female	695	(41)
Age at HCT [years]	Median	61	
	IQR	53 – 66	
	Range	18 – 83	
Registry	EBMT	1208	(71)
	CIBMTR	496	(29)
Disease	MDS	1076	(63)
	MDS/MPD	155	(9)
	sAML, tAML	473	(28)
Disease Risk	Intermediate	706	(41)
	High	987	(58)
	Very High	11	(1)
Karnofsky Status	90-100%	1075	(63)
	80%	428	(25)
	≤80%	157	(9)
	*Missing information*	44	(3)
T-cell Depletion	No T-cell depletion	590	(35)
	Anti-thymocyte globulin	946	(56)
	Alemtuzumab	145	(9)
	*ex vivo* T cell depletion	23	(1)
Conditioning Intensity	Myeloablative	522	(31)
	Reduced	949	(56)
	Non-myeloablative	191	(11)
	*Missing information*	42	(2)
Conditioning	TBI-based	235	(14)
	Chemotherapy-based	1268	(74)
	*Missing information*	201	(12)
Donor Age [years]	Median	28	
	IQR	23 – 36	
	Range	18 – 60	
HLA-Match	10/10 matched	1346	(79)
	9/10 (DQB1 mm)	71	(4)
	9/10 (A,B,C or DRB1 mm)	277	(16)
	≤8/10 matched	10	(1)
Patient–Donor Sex Constellation	Male–male	767	(45)
	Male–female	242	(14)
	Female–male	439	(26)
	Female–female	256	(15)
Patient–Donor CMV Serostatus	Negative–negative	540	(32)
	Negative–positive	114	(7)
	Positive–negative	545	(32)
	Positive–positive	477	(28)
	*Missing information*	28	(2)
Graft Source	PBSC	1587	(93)
	Bone Marrow	117	(7)
Year of HCT	2008 – 2012 2013 2014 2015 2016 2017	11 140 408 480 491 174	(1) (8) (24) (28) (29) (10)

IQR, interquartile range; EBMT, European Society for Blood and Marrow Transplantation; CIBMTR, Center for International Blood and Marrow Transplant Research; AML, acute myeloid leukemia; sAML, secondary AML; tAML, therapy-related acute myeloid leukemia; MDS, myelodysplastic syndrome; HLA, human leukocyte antigen; mm, mismatch; CMV, cytomegalovirus; TBI, total body irradiation; PBSC, peripheral blood stem cells; HCT, hematopoietic cell transplantation.

For the whole cohort, 2-year probabilities were 48% (95%-CI: 46–51%) for OS, 42% (95%-CI: 39–44%) for EFS, 29% (95%-CI: 27–32%) for relapse incidence and 29% (95%-CI: 27–32%) for Non-Relapse Mortality. In total, 451 relapses and 450 non-relapse deaths were recorded. Altogether, 780 deaths were reported, including 330 deaths after relapse.

### KIR—Ligand Models

**Figure 1:**
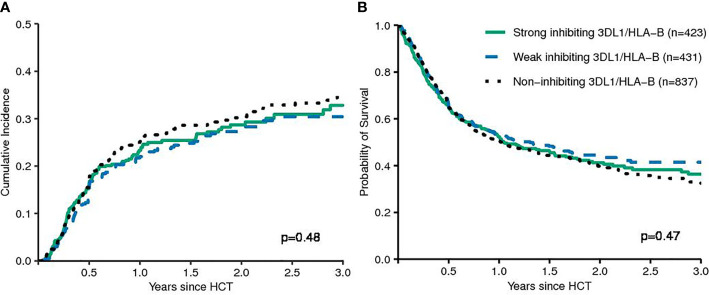
Patients were grouped by their donors’ *KIR3DL1/HLA-B* subtype combinations (strong inhibiting versus non-inhibiting/weak inhibiting) in terms of relapse incidence **(A)** and event-free survival **(B)**. The p-values have been calculated by the Gray Test (CIR) and the log-rank test (EFS).

**Figure 2:**
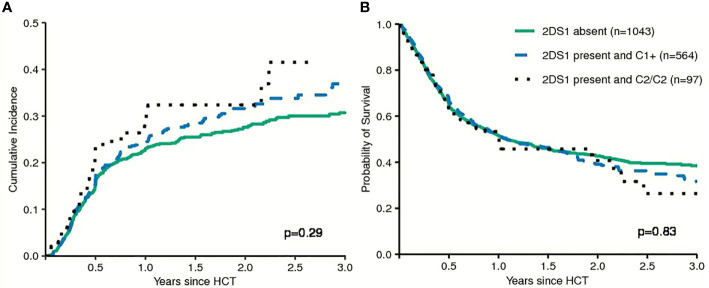
Patients were grouped by their donors' *KIR2DS1* status (activating versus non-activating) in terms of Relapse Incidence **(A)** and Event-free Survival **(B)**. The p-values have been calculated by the Gray Test (Cumulative Incidence of Relapse) and the log-rank test (Event-free Survival).

First, models integrating information on activating or non-activating KIR2DS1 and various degrees of KIR3DL1-mediated inhibition were tested for their ability to predict the risk of relapse and EFS (12, 13). Basically, these models were built on the idea that strong inhibitory KIR-ligand interactions increase the risk of relapse while activating KIR-ligand interactions reduce it. The results of multivariable modelling are summarized in **Table 2**. Detailed information on the set of factors used for risk adjustment is provided in **Supplemental Table 1**. No significant differences for the risk of relapse were found for the respective *KIR3DL1/HLA-B* subtype combinations and KIR2DS1/C1C2 epitope combinations. Notably, in contrast to the original publications for these models, neither patients whose donors had non-inhibiting KIR3DL1-Ligand interaction (HR 1.08, 95%CI 0.86–1.36; p=.5) nor patients with activating KIR2DS1-Ligand interaction (HR 1.11, 95%CI 0.87–1.16; p=0.9) showed a lower risk of relapse. Cumulative incidence curves for relapse and Kaplan-Meier plots for EFS are shown in **Figures 1** and **2** for the respective groups of patients.

**Table 2:** Impact of donor *KIR* genotype models on relapse incidence and event-free survival.

Classifier	N	%	Relapse Incidence		Event-free Survival		Endpoint - Power
			HR (95%-CI)	p	HR (95%-CI)	p	HR for CIR; 1-β (α=0.05)
***KIR3DL1/HLA-B* subtype combinations**							
Strong inhibiting *KIR3DL1*	423	(25)	1		1		
Weak-inhibiting *KIR3DL1*	431	(25)	0.92 (0.71–1.21)	0.6	0.92 (0.76–1.11)	0.4	HR 0.75 (13); 0.64
Non-inhibiting *KIR3DL1*	837	(49)	1.08 (0.86–1.36)	0.5	1.03 (0.87–1.21)	0.7	HR 0.84 (13); 0.71
***KIR2DS1/*C1C2 epitope combinations**							
*KIR2DS1* neg	1043	(61)	1		1		
*KIR2DS1* pos/C1+	564	(33)	1.11 (0.91–1.35)	0.3	1.00 (0.87–1.16)	0.9	HR 0.69 (12); 0.95
*KIR2DS1* pos/C2/C2	97	(6)	1.29 (0.87–1.90)	0.2	1.08 (0.81–1.44)	0.6	HR 1.51 (12); 0.50
**KIR haplotype motif-based models** (10)							
*Cen A/A*	798	(47)	1		1		
*Cen A/B*	735	(43)	1.17 (0.96–1.43)	0.13	1.05 (0.92–1.21)	0.5	HR 0.87 (10); 0.28
*Cen B/B*	171	(10)	1.19 (0.88–1.62)	0.3	0.86 (0.68–1.08)	0.2	HR 0.34 (10); 1.00
*Tel A/A*	1006	(59)	1		1		
*Tel A/B*	619	(36)	1.06 (0.87–1.29)	0.6	1.01 (0.87–1.16)	0.9	HR 0.70 (10); 0.95
*Tel B/B*	79	(5)	1.29 (0.85–1.96)	0.2	1.07 (0.79–1.46)	0.7	HR 0.52 (10); 0.82
“neutral” KIR-score (*B ≤* 1)	1173	(69)	1		1		
“better” KIR-score (any other)	360	(21)	1.13 (0.90–1.43)	0.3	1.01 (0.85–1.19)	0.9	HR 0.70 (10); 0.96
“best” KIR-score (*Cen-B/B)*	171	(10)	1.14 (0.85–1.53)	0.4	0.84 (0.66–1.05)	0.1	HR 0.52 (10); 1.00
**Additive inhibitory KIR - Ligand Model** (24)							
Count Functional iKIR (cont.)	1704	(100)	0.91 (0.80–1.03)	0.12	0.90 (0.83–0.99)	0.02	^§^not applicable
Count Functional iKIR ≤1	444	(26)	1		1		
Count Functional iKIR >1	1260	(74)	0.76 (0.61–0.93)	0.01	0.80 (0.69–0.93)	0.004	
Inhibitory Score (cont.)	1704	(100)	0.96 (0.86–1.07)	0.4	0.95 (0.88–1.02)	0.15	
Inhibitory Score (Cutoff ≤ 1.75)	711	(42)	1		1		
Inhibitory Score (Cutoff>1.75)	993	(58)	0.92 (0.76–1.11)	0.4	0.87 (0.76–0.99)	0.04	
**Additive inhibitory/activating KIR – Ligand Model** (25)							
w-KIR-Score (cont.)	1704	(100)	1.04 (0.87–1.24)	0.6	1.01 (0.89–1.14)	0.9	HR 0.44 (25); 1.00
IM-KIR-Score (cont.)	1704	(100)	1.00 (0.85–1.17)	1.0	1.00 (0.89–1.11)	1.0	HR 0.44 (25); 1.00

N, number; HR, hazard ratio; p, p-value; neg, negative; pos, positive; cen, centromeric; tel, telomeric; iKIR, inhibitory Killer Immunoglobulin like Receptors; cont, continuous; w-KIR-Score, weighted KIR-Score; IM-KIR-Score, inhibitory-missing KIR-ligand Score CIR, cumulative incidence of relapse; Hazard ratios were calculated in (cause-specific) multivariable Cox regression models stratified by registry (CIBMTR or EBMT), and adjusted for patient age, donor age, diagnosis, disease risk index, Karnofsky performance status, conditioning intensity, T-cell depletion, sex match, CMV match, HLA-match, and stem cell source. The p-value of the Wald Test is reported. The power was calculated according to Schoenfeld’s formula (29).

^§^The various models of Boelen et al. have not been evaluated in the context of allogeneic HCT. No estimates for the effect size were available. Therefore, we did not perform power assessments for this model.

### Haplotype Motif-Based Prediction

**Figure 3:**
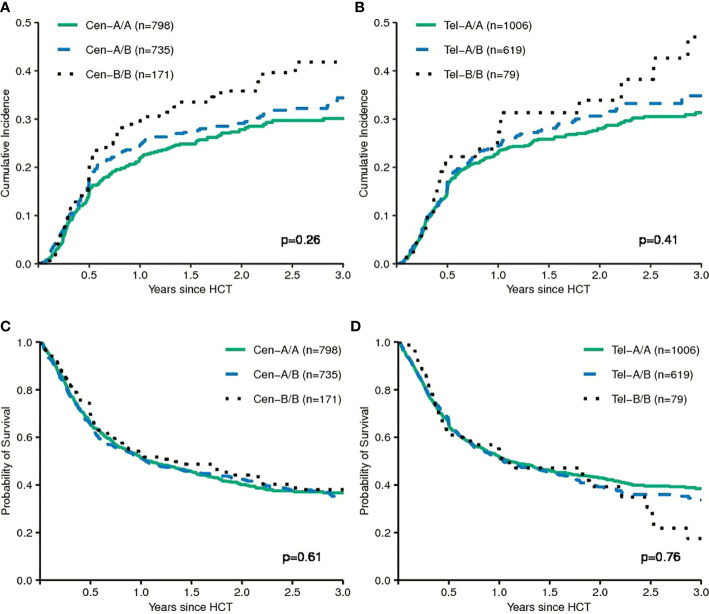
The upper panels show the cumulative incidences of relapse of patients grouped according to centromeric KIR motifs **(A)** and grouped according to telomeric KIR motifs **(B)**. The lower panels show event-free survival of patients grouped according to centromeric KIR motifs **(C)** and grouped according to telomeric KIR motifs **(D)**. The p-values have been calculated by the Gray Test (Cumulative Incidence of Relapse) and the log-rank test (Event-free Survival).

Next, we tested models which utilize information on the different composition of *KIR* haplotypes with respect to activating and inhibitory *KIR* genes. The *KIR A* haplotypes are more conserved and contain largely inhibitory *KIR* genes, whereas the *B* haplotypes display greater variation in gene content and may include a variety of activating receptors. Some studies suggested that patients whose donors had two *B* haplotypes had a lower risk of relapse compared to patients whose donors had two *A* haplotypes (9–11). In our data we did not find a significant impact of telomeric or centromeric *KIR* haplotype A versus *B* motifs. Compared to patients, whose donors had centromeric *KIR A/A* motifs, patients, whose donors had centromeric *KIR B/B* motifs, even tended to a greater risk of relapse (HR 1.19, 95%CI 0.88–1.62; p=.3). Cumulative incidence curves for relapse and Kaplan-Meier plots for EFS are shown in **Figure 3** for the respective grouping of patients according to centromeric donor *KIR* haplotypes. In contrast to the model, we also found a trend for a greater risk of relapse (HR 1.29, 95%CI 0.85–1.96; p=0.2) for patients with telomeric *KIR B/B* donors compared to telomeric *KIR A/A* donors. **Table 2** summarizes the results.

### Additive Models

Finally, we tested models which integrate information on inhibitory or activating KIR-ligand interactions using a scoring system. Boelen et al. developed an inhibitory score which takes information on functional inhibitory KIR-ligand interactions into account and demonstrated uniform effects of the score on the progression of HIV, HCV, and HTLV infections (24). In the context of these viral infections, high inhibitory scores were mainly associated with protective effects. The weighted inhibitory score did not predict the risk of relapse in our study. However, the functional inhibitory KIR count was significantly associated with the risk of relapse (HR 0.76, 95%CI 0.61–0.93; p=0.01) and EFS (HR 0.8, 95%CI 0.69–0.93; p=0.004), when dichotomized at a cutoff of 1. This comparison indicated that donor genotypes encoding more functionally relevant inhibitory *KIR* were associated with a lower risk of relapse.

The score proposed by Krieger et al. integrates information on potential inhibitory KIR-ligand interactions and activating KIR-ligand interactions (25). Neither the simple score nor the weighted score predicted the risk of relapse or EFS in our cohort.

### Subgroup Analyses

We also calculated KIR haplotype motif-based models for four major subgroups: patients with secondary AML, patients with MDS, patients who had received myeloablative conditioning and patients who had received non-myeloablative or reduced-intensity conditioning. In a series of exploratory analyses we tested the main haplotype motif-based classifications for these subgroups. Results are shown in **Supplemental Table 2A**. The effects of centromeric and telomeric *KIR* haplotype *B/B* motifs compared to haplotype *A/A* motifs pointed in different directions for patients with secondary AML (HR 0.7, 95%–CI 0.4–1.33) and patients with MDS (HR 1.48, 95%–CI 1.03–2.11). The estimated effects for donor *KIR* haplotype motifs for patients who had received reduced-intensity or non-myeloablative conditioning also differed slightly from patients who had received myeloablative conditioning. Further, we performed subgroup analyses in patients with at least one C1 epitope, thereby excluding C2/C2 patients, which showed essentially in the same pattern of results (**Supplemental Table 2B**).

## Discussion

We investigated the potential of donor *KIR* genotype information to predict the risk of relapse after HLA-compatible unrelated allogeneic HCT in a large cohort of patients with secondary acute myeloid leukemia (sAML) or a myelodysplastic syndrome (MDS). To date, few studies have analysed the impact of *KIR* genotype on the course of the disease for patients with MDS or sAML after HLA-compatible alloHCT (6, 31, 32). Since patient numbers in these studies were small, no meaningful subgroup analyses for patients with MDS or sAML were performed. Therefore, our results should be discussed in the context of studies on patients with AML.

This study was designed as a validation study for the *KIR2DS1/KIR3DL1*-based prediction model in patients with MDS and sAML. This model claims that the risk of relapse could be reduced by increasing the activating potential through preferentially selecting unrelated donors with activating *KIR2DS1* and by minimizing the inhibitory potential mediated through selecting *KIR3DL1*/*HLA-B* subtype combinations with minimal or no inhibitory potential (12, 13). We were not able to validate this model for patients with MDS or sAML. Although not statistically significant, unrelated donor-patient pairs with activating *KIR2DS1* even had a slightly higher actual risk of relapse indicated by a hazard ratio of 1.11 (Wald test, p=0.3) compared to patients whose donors where *KIR2DS1* negative. Contrary to the previously observed effects, donor-patient pairs with non-inhibiting *KIR3DL1/HLA-B* subtype combinations also had the highest actual risk of relapse with a hazard ratio of 1.08 (Wald test, p=0.5) in multivariable cause-specific Cox regression modelling (see **Table 2** and **Figure 1**). In summary, our study did not align with the pattern of results originally reported. In a recently published study where we attempted to validate the proposed model for patients with AML, the observed data also failed to confirm the proposed prediction model (23). Together, in these two studies data from 3926 patients with AML or MDS were analysed whose HLA-compatible unrelated donors had been typed for *KIR* genes at the allele-level. However, it should be noted that patient characteristics and transplant procedures of the original cohorts differed substantially compared to the patients whose data were analysed here. In our contemporary validation studies the median patient age was approximately 20 years higher compared to the original cohort and more patients were matched for HLA-A, -B, -C, -DRB1, and DQB1 with their unrelated donors. Patients had predominantly received chemotherapy-based reduced-intensity conditioning instead of Total Body Irradiation (TBI)-based myeloablative conditioning, ATG for the prophylaxis of Graft versus Host Disease (GvHD) instead of *ex vivo* T-cell depletion, and had been transplanted with Peripheral Blood Stem Cells (PBSC) instead of bone marrow as graft source (12, 13). All factors reflect changes in clinical practice between the past cohorts and this contemporary cohort. Although little is known about the impact of different procedural choices on NK cell reconstitution after alloHCT, these factors could change NK cell mediated alloreactivity.

In order to comprehensively re-assess the work on outcome prediction after alloHCT based on the donor *KIR* genotype, we also evaluated alternative models. Genotypes can be categorized based on the absence and presence of certain activating and inhibiting *KIR* genes according to their content of centromeric and telomeric haplotype *A* and *B* motifs (33). Haplotype *B* motifs occur at lower frequencies in the Caucasian population and harbour more activating *KIR* genes (18). In larger studies, the presence and number of centromeric haplotype B motifs in the donor genome have been associated with a reduced risk of relapse after HLA-compatible unrelated allogeneic transplantation for AML (9–12). An updated and extended analysis was recently published by Weisdorf et al. (34). In a large contemporary cohort of AML patients the authors were able to demonstrate a beneficial impact of *KIR* haplotype *B* donors in the subgroup of C1/C1 or C1/C2 patients who had received reduced-intensity conditioning. In a strict sense, the attempt to validate their findings on the impact of haplotype B motifs on the risk of relapse after myeloablative conditioning therefore failed (11). Other groups have reported conflicting results which even indicated a protective effect of *KIR* haplotype *A* from smaller registry studies (35–37). In this study, we did not find a protective effect in univariable and multivariable comparisons for centromeric or telomeric *KIR* haplotype *B* versus *A* motifs in a contemporary cohort of patients with MDS of equal size (see **Table 2** and **Figure 3**). Even, when we restricted the analyses to those subgroups, who had shown the greatest effects of *KIR* haplotype *B* donors, i.e. C1-positive patients who had received reduced-intensity conditioning, we did not observe significant effects in the predicted direction (**Supplemental Table 2B**).

Of note, in our exploratory analyses we observed opposite effects of donor *KIR* haplotype *B* motifs for patients with MDS versus secondary AML (see **Supplemental Table 2A**). For example, in MDS donor centromeric *KIR* haplotype *B* motifs appeared to be disadvantageous (HR, 1.48, p=0.03 for *KIR cen B/B* versus *KIR cen A/A*) whereas in secondary AML it appeared to be advantageous (HR, 0.73, p=0.3 for *KIR cen B/B* versus *KIR cen A/A*). Stringaris et al. reported that patients with MDS and *KIR* haplotype *A* show a more aggressive course of their disease (38). This would be in line with our results for patients with secondary AML but not with MDS. Nevertheless, we would like to caution against over-interpreting this signal. Reduced expression of HLA class I molecules which represent important KIR-ligands was not different in a recent study analysing samples from MDS and secondary AML for features which might explain escape from immune surveillance (39). Moreover, the group of patients with secondary AML was relatively small (N=473) in our study compared to the group of patients with MDS (N=1231) and it is possible that unknown confounders produced this difference. On the other hand, the different effect directions of *KIR* haplotypes in different types of diseases require careful consideration for future analyses.

Results from the exploratory subgroup analyses also revealed some differences for the estimated effects of *KIR* haplotype motifs in the context of reduced-intensity or non-myeloablative conditioning versus myeloablative conditioning, albeit not in a systematic way. Most important, the observed differences were not in line with what has been reported recently by Weisdorf et al. (34) and may be due to random effects.

The current assignment of the *putative KIR* haplotypes *A* and *B* as proposed by Uhrberg et al. in 1997 does not integrate allele-level information. With *KIR* genotyping at allele-level, *KIR* gene haplotypes based only on absence/presence information appear over-simplistic and may no longer represent a suitable grouping algorithm (40–42). While we found no impact of the classification into putative *KIR* haplotypes *A* and *B*, improved assignments based on *KIR* allele groups have the potential to reflect functional information of distinct *KIR* haplotypes more precisely.

A logical extension to the investigation of single KIR KIR-ligand combinations is creating additive models which integrate information on multiple *KIR* genes together with the corresponding ligands. One such additive model had been composed to predict NK-cell mediated control of chronic viral infections. The score is based on the number of functional inhibitory KIRs and predicts the progression of HIV, HCV, and HTLV-1 infections (24). The crude score of this model predicted the risk of relapse and EFS also in our data (see **Table 2**). In multivariable Cox regression analyses unrelated patient-donor pairs with an inhibitory KIR count of greater than 1 had a 24% lower risk of relapse (Wald-test, p=0.01) and a 20% lower risk (Wald-test, p=0.004) for events defining EFS compared to the patients with a single or no functional inhibitory KIR KIR-ligand combination. This association suggested that NK cells which were educated by multiple KIR–KIR–ligand interactions exerted stronger NK-mediated alloreactivity. Downregulation of HLA class I molecules on the malignant target cells would then be a necessary trigger for activation. Although down-regulation of class I molecules to escape T-cell attack is a common feature of cells infected by viruses and cancer cells, this mechanism was not found to be a major immune escape strategy after alloHCT. Recent data suggest that the primary immune escape mechanism after alloHCT starts by down-regulation of HLA-class II molecules (43, 44). The observed beneficial impact of a higher inhibitory KIR count is therefore not supported by the currently-favoured concept of immune escape of malignant cells after allogeneic transplantation (45). On the other hand, due to a lack of a humanized animal model which recapitulates graft versus leukemia effects, the basic biological principles of NK cell mediated allo-reactivity remain uncertain. Thus, the jury is still out, on whether donors with more or less functionally inhibitory KIRs may exert stronger graft-versus leukemia reactions after HLA-compatible unrelated alloHCT. The testing of integrated scores which reflect a conclusive biological concept in large registry studies may further inform our understanding of NK biology and more research in this regard is warranted. However, since the functionally inhibitory KIR count was tested here as part of a series of exploratory analyses without keeping stringent control of the family-wise type I error rate, we do not recommend application of the functionally inhibitory KIR count for donor selection in the context of stem cell transplantation based on the current data.

From a conceptual point of view the integration of information on donor KIRs and patient KIR-ligands in one unifying score is appealing. Very likely, an optimal score for the prediction of relapse after matched unrelated donor alloHCT will be complex and may contain first- and second-order interactions. Weights for single factors must be defined carefully. Mathematically step-functions may be more appropriate to predict threshold-dependent NK cell activation or inhibition than linear functions. However, given the stochastic expression of KIRs on NK cells composing the individual repertoire, the breadth of a potential NK response may also be impacted. This would weigh an argument in favour of linear or monotonous relations. To address these challenges in model-building, machine learning algorithms may become necessary tools. Given recent successes in the use of artificial intelligence in medical science and the complexity of *KIR* genetics and NK biology in the transplant context, this area of research appears to be especially appealing for the application of these new techniques (46).

Critical resources required to answer the question on whether the donor *KIR* genotype can be used to predict patient outcome after HLA-compatible unrelated alloHCT, are i) access to donor samples donated for research at biobanks, ii) collaborative efforts to pull together large datasets and sample sizes necessary to apply machine learning tools or other complex statistical models, iii) access to affordable allele-level KIR typing, iv) stringent statistical testing strategies to keep control of the family-wise type I error and to validate findings in independent datasets, and finally v) active research groups committed to advancing the understanding of NK biology in the context of alloHCT (47, 48). The European Society for Blood and Marrow Transplantation (EBMT), the Center for International Blood and Marrow Transplant Research (CIBMTR), the National Marrow Donor Program (NMDP) and DKMS are committed to answering this question and can provide critical resources. A large collaborative effort of these institutions to come to a conclusive answer is currently underway.

In summary, despite availability of *KIR* genotype information for more than 3 million potential stem cell donors, no *KIR*-based algorithm for unrelated donor selection has entered clinical practice. After more than 20 years of research, the impact of donor *KIR* genotype information on the outcome after unrelated donor alloHCT is still not clear. This large study of patients with MDS and secondary AML adds to the growing body of data by showing that the KIR KIR-ligand combinations, *KIR2DS1*-C2 and *KIR3DL1*-Bw4(80I/T) and putative haplotype motif based models have no impact on the risk of relapse and mortality after unrelated donor alloHCT. However, with larger studies and intergroup collaborations on the horizon, high-throughput allelic resolution of *KIR* genes at hand and increasing experience in dealing with high-dimensional data, the chances are good that the question on whether *KIR* genotype information can be used for donor selection will be resolved in the next couple of years.

## Author's Note

This was a joint study of the Chronic Malignancies Working Party of the European Society for Blood and Marrow Transplantation (EBMT) and the Center for International Blood and Marrow Transplant Research (CIBMTR).

## Data Availability Statement

The datasets presented in this article are not readily available because the medical data belongs to the EBMT and the CIBMTR. Generated datasets can be requested by the corresponding author conditional on the approval of EBMT and CIBMTR. Requests to access the datasets should be directed to johannes.schetelig@ukdd.de.

## Ethics Statement

The studies involving human participants were reviewed and approved by Ethical Committee of the Technische Universität Dresden. Access to medical data was approved by the Review Boards of the Chronic Malignancies Working Party of EBMT and the Immunobiology Working Committee of the CIBMTR and the National Marrow Donor Program Institutional Review Board. All patients and donors gave written informed consent to the use of samples and medical data for medical research.

## Author Contributions

JS, HB, FH, AS, and MR designed the study. JS, MK SS, MG, BB, FO, VP, PL, NS, PH, SL, NK, KH, IY-A, and MR contributed medical data. HB, LK, MK, LW, and CM contributed to the different levels of the processing of genetic and medical data. JS, MK, HB, and LW performed the statistical analysis. JS and HB wrote the manuscript. All authors interpreted and discussed the results, and reviewed and approved the manuscript. All authors contributed to the article and approved the submitted version.

## Funding

This study was enabled by DKMS gGmbH who financed the donor KIR typing and supported this study with know-how and logistics. No funding from commercial entities was received for this study. The CIBMTR is supported primarily by Public Health Service U24CA076518 from the National Cancer Institute (NCI), the National Heart, Lung and Blood Institute (NHLBI), and the National Institute of Allergy and Infectious Diseases (NIAID); U24HL138660 from NHLBI and NCI; R21HL140314, and U01HL128568 from the NHLBI; HHSH250201700006C, SC1MC31881-01-00, and HHSH250201700007C from the Health Resources and Services Administration (HRSA); and N00014-18-1-2850, N00014-18-1-2888, and N00014-20-1-2705 from the Office of Naval Research. Additional federal support is provided by P01CA111412, R01CA152108, R01CA215134, R01CA218285, R01CA231141, R01HL126589, R01AI128775, R01HL129472, R01HL130388, R01HL131731, U01AI069197, U01AI126612, and BARDA. Support is also provided by Be the Match Foundation, Boston Children’s Hospital, Dana Farber, Japan Hematopoietic Cell Transplantation Data Center, St. Baldrick’s Foundation, the National Marrow Donor Program, the Medical College of Wisconsin and from the following commercial entities: AbbVie; Actinium Pharmaceuticals, Inc.; Adaptive Biotechnologies; Adienne SA; Allovir, Inc.; Amgen, Inc.; Anthem, Inc.; Astellas Pharma US; AstraZeneca; Atara Biotherapeutics, Inc.; bluebird bio, Inc.; Bristol Myers Squibb Co.; Celgene Corp.; Chimerix, Inc.; CSL Behring; CytoSen Therapeutics, Inc.; Daiichi Sankyo Co., Ltd.; Gamida-Cell, Ltd.; Genzyme; GlaxoSmithKline (GSK); HistoGenetics, Inc.; Incyte Corporation; Janssen Biotech, Inc.; Janssen Pharmaceuticals, Inc.; Janssen/Johnson & Johnson; Jazz Pharmaceuticals, Inc.; Kiadis Pharma; Kite Pharma; Kyowa Kirin; Legend Biotech; Magenta Therapeutics; Mallinckrodt LLC; Medac GmbH; Merck & Company, Inc.; Merck Sharp & Dohme Corp.; Mesoblast; Millennium, the Takeda Oncology Co.; Miltenyi Biotec, Inc.; Novartis Oncology; Novartis Pharmaceuticals Corporation; Omeros Corporation; Oncoimmune, Inc.; Orca Biosystems, Inc.; Pfizer, Inc.; Phamacyclics, LLC; Regeneron Pharmaceuticals, Inc.; REGiMMUNE Corp.; Sanofi Genzyme; Seattle Genetics; Sobi, Inc.; Takeda Oncology; Takeda Pharma; Terumo BCT; Viracor Eurofins; and Xenikos BV. The views expressed in this article do not reflect the official policy or position of the National Institute of Health, the Department of Navy, the Department of Defense, Health Resources and Services Administration (HRSA), or any other agency of the U.S. Government.

## Conflict of Interest

The DKMS Life Science Laboratory (VL, CM, AS) implemented the KIR genotyping as part of the upfront genotyping profile for volunteers enrolled into the DKMS and offers KIR genotyping also for external customers.

The remaining authors declare that the research was conducted in the absence of any commercial or financial relationships that could be construed as a potential conflict of interest.
