# Corrigendum: Haplotype Motif-Based Models for KIR-Genotype Informed Selection of Hematopoietic Cell Donors Fail to Predict Outcome of Patients With Myelodysplastic Syndromes or Secondary Acute Myeloid Leukemia

**DOI:** 10.3389/fimmu.2021.813838

**Published:** 2021-12-21

**Authors:** Johannes Schetelig, Henning Baldauf, Linda Koster, Michelle Kuxhausen, Falk Heidenreich, Liesbeth C. de Wreede, Stephen Spellman, Michel van Gelder, Benedetto Bruno, Francesco Onida, Vinzenz Lange, Carolin Massalski, Victoria Potter, Per Ljungman, Nicolaas Schaap, Patrick Hayden, Stephanie J. Lee, Nicolaus Kröger, Kathy Hsu, Alexander H. Schmidt, Ibrahim Yakoub-Agha, Marie Robin

**Affiliations:** ^1^ Medizinische Klinik und Poliklinik I, University Hospital Dresden, Dresden, Germany; ^2^ DKMS Clinical Trials Unit, Dresden, Germany; ^3^ EBMT Data Office Leiden, Leiden, Netherlands; ^4^ Center for International Blood and Marrow Transplant Research, Minneapolis, MN, United States; ^5^ Leiden University Medical Center, Department of Biomedical Data Sciences, Leiden, Netherlands; ^6^ Maastricht University Medical Center, Department of Internal Medicine, Maastricht, Netherlands; ^7^ A.O.U. Citta della Salute e della Scienza di Torino, Turin, Italy; ^8^ Fondazione IRCCS Ca’Granda Ospedale Maggiore Policlinico, University of Milan, Milan, Italy; ^9^ DKMS Life Science Lab, Dresden, Germany; ^10^ GKT School of Medicine, London, United Kingdom; ^11^ Karolinska University Hospital and Karolinska Institutet, Stockholm, Sweden; ^12^ Radboud University Medical Centre, Nijmegen, Netherlands; ^13^ St. James’s Hospital, Dublin, Ireland; ^14^ Fred Hutchinson Cancer Research Center, Seattle, WA, United States; ^15^ University Hospital Eppendorf, Hamburg, Germany; ^16^ Memorial Sloan Kettering Cancer Center, New York & Scientific Director, CIBMTR Immunobiology Working Committee, New York City, NY, United States; ^17^ CHU de Lille, LIRIC, INSERM U995, Universite´ de Lille, Lille, France; ^18^ Hopital Saint-Louis, APHP, Universite´ de Paris, Paris, France

**Keywords:** KIR, KIR2DS1, KIR3DL1, hematopoietic stem cell transplantation, donor selection, unrelated donor

There is an error in the Funding statement. The CIBMTR support erroneously included the grant number OT3HL147741 that is not associated with this work.

The authors apologize for this error and state that this does not change the scientific conclusions of the article in any way. The original article has been updated.

## Publisher’s Note

All claims expressed in this article are solely those of the authors and do not necessarily represent those of their affiliated organizations, or those of the publisher, the editors and the reviewers. Any product that may be evaluated in this article, or claim that may be made by its manufacturer, is not guaranteed or endorsed by the publisher.

