# Supplementary material for: Haplotype Motif-Based Models for KIR-Genotype Informed Selection of Hematopoietic Cell Donors Fail to Predict Outcome of Patients With Myelodysplastic Syndromes or Secondary Acute Myeloid Leukemia
Source: Front Immunol. 2021 Jan 19;11:584520. doi: 10.3389/fimmu.2020.584520 (PMC7851088; doi:10.3389/fimmu.2020.584520)
Supplement: Supplementary file 1 [file DataSheet_1.zip › Table S1.DOC]

**Supplemental Tables**

**Table S1. Multivariable Cox Regression modelling with the classifier for donor *KIR3DL1/HLA-B*** subtypes

| **Variable** | | **Overall Survival** | | **Event-Free Survival** | | | | | **Relapse** | | | | **Non-Relapse Mortality** | | |
| --- | --- | --- | --- | --- | --- | --- | --- | --- | --- | --- | --- | --- | --- | --- | --- |
|  |  | **HR (95% CI)** | **p-value** | **HR** | **(95% CI)** | **p-value** | | | **HR (95% CI)** | **p-value** | | | **HR (95% CI)** | | **p-value** |
| *3DL1/HLA-B* subtypes | Strong inhibiting | 1 |  | 1 | | |  | 1 | | |  | 1 | |  | |
|  | Weak inhibiting | 0.97 (0.79-1.18) | 0.8 | 0.92 (0.76-1.11) | | | 0.4 | 0.93 (0.71-1.21) | | | 0.6 | 0.91 (0.70-1.18) | | 0.5 | |
|  | Non-inhibiting | 1.06 (0.89-1.26) | 0.5 | 1.03 (0.87-1.21) | | | 0.7 | 1.08 (0.86-1.36) | | | 0.5 | 0.98 (0.78-1.23) | | 0.9 | |
| Patient age | (per 10 years) | 1.14 (1.06-1.23) | <0.001 | 1.11 (1.04-1.19) | | | 0.002 | 1.05 (0.96-1.16) | | | 0.3 | 1.18 (1.07-1.30) | | 0.001 | |
| Donor age | (per 10 years) | 1.15 (1.07-1.24) | <0.001 | 1.12 (1.05-1.21) | | | 0.001 | 1.09 (0.99-1.21) | | | 0.08 | 1.15 (1.04-1.27) | | 0.005 | |
| Diagnosis | MDS. MDS/MPN | 1 |  | 1 | | |  | 1 | | |  | 1 | |  | |
|  | sAML. tAML | 1.28 (1.07-1.52) | 0.01 | 1.34 (1.14-1.58) | | | <0.001 | 1.83 (1.46-2.29) | | | <0.001 | 0.99 (0.78-1.25) | | 0.9 | |
| Disease Risk  Index | low or intermediate | 1 |  | 1 | | |  | 1 | | |  | 1 | |  | |
|  | high or very high | 1.26 (1.08-1.48) | 0.004 | 1.32 (1.14-1.54) | | | <0.001 | 1.50 (1.21-1.86) | | | <0.001 | 1.18 (0.96-1.46) | | 0.12 | |
| Karnofsky performance  status | 90 or 100 | 1 |  | 1 | | |  | 1 | | |  | 1 | |  | |
|  | 80 | 1.36 (1.15-1.60) | <0.001 | 1.30 (1.12-1.52) | | | 0.001 | 1.25 (1.00-1.55) | | | 0.050 | 1.35 (1.09-1.68) | | 0.01 | |
|  | <80 | 1.52 (1.21-1.91) | <0.001 | 1.42 (1.14-1.76) | | | 0.002 | 1.29 (0.95-1.77) | | | 0.11 | 1.56 (1.15-2.11) | | 0.004 | |
|  | missing information | 0.87 (0.52-1.46) | 0.6 | 0.88 (0.54-1.41) | | | 0.6 | 1.23 (0.67-2.28) | | | 0.5 | 0.59 (0.28-1.26) | | 0.2 | |
| Conditioning Intensity | Myeloablative | 1 |  | 1 | | |  | 1 | | |  | 1 | |  | |
|  | Reduced | 0.84 (0.71-1.00) | 0.047 | 0.88 (0.75-1.03) | | | 0.11 | 0.96 (0.76-1.21) | | | 0.7 | 0.81 (0.64-1.01) | | 0.06 | |
|  | Non-myeloablative | 1.06 (0.82-1.37) | 0.6 | 1.04 (0.82-1.33) | | | 0.7 | 1.12 (0.80-1.58) | | | 0.5 | 0.98 (0.69-1.38) | | 0.9 | |
|  | missing information | 0.68 (0.42-1.12) | 0.1 | 0.66 (0.41-1.07) | | | 0.09 | 0.73 (0.38-1.40) | | | 0.3 | 0.59 (0.28-1.23) | | 0.2 | |
| T-cell depletion | No T-cell depletion | 1 |  | 1 | | |  | 1 | | |  | 1 | |  | |
|  | Anti-Thymocyte Globulin | 1.32 (1.11-1.56) | 0.001 | 1.31 (1.12-1.54) | | | 0.001 | 1.14 (0.92-1.42) | | | 0.2 | 1.53 (1.22-1.93) | | <0.001 | |
|  | Alemtuzumab/Ex-vivo | 1.28 (0.97-1.69) | 0.1 | 1.23 (0.95-1.59) | | | 0.1 | 0.97 (0.66-1.42) | | | 0.9 | 1.54 (1.08-2.20) | | 0.02 | |
| Sex match (patient/donor) | male/male | 1 |  | 1 | | |  | 1 | | |  | 1 | |  | |
|  | male/female | 1.02 (0.82-1.27) | 0.9 | 1.01 (0.82-1.23) | | | 0.9 | 0.91 (0.68-1.21) | | | 0.5 | 1.11 (0.84-1.47) | | 0.5 | |
|  | female/male | 1.03 (0.87-1.23) | 0.7 | 1.06 (0.90-1.25) | | | 0.5 | 1.01 (0.80-1.28) | | | 0.9 | 1.12 (0.89-1.42) | | 0.3 | |
|  | female/female | 1.02 (0.83-1.27) | 0.8 | 1.00 (0.82-1.22) | | | 1.0 | 0.87 (0.65-1.16) | | | 0.3 | 1.14 (0.86-1.51) | | 0.4 | |
| CMV match (patient/donor) | negative/negative | 1 |  | 1 | | |  | 1 | | |  | 1 | |  | |
|  | negative/positive | 1.07 (0.79-1.44) | 0.7 | 1.04 (0.78-1.37) | | | 0.8 | 1.13 (0.78-1.65) | | | 0.5 | 0.92 (0.60-1.41) | | 0.7 | |
|  | positive/negative | 1.11 (0.93-1.33) | 0.3 | 1.02 (0.86-1.20) | | | 0.8 | 0.95 (0.75-1.20) | | | 0.7 | 1.09 (0.86-1.39) | | 0.5 | |
|  | positive/positive | 1.01 (0.84-1.22) | 0.9 | 0.93 (0.78-1.11) | | | 0.4 | 0.86 (0.67-1.10) | | | 0.2 | 1.01 (0.79-1.29) | | 0.9 | |
|  | missing information | 1.44 (0.84-2.47) | 0.2 | 1.43 (0.87-2.37) | | | 0.2 | 1.44 (0.72-2.88) | | | 0.3 | 1.41 (0.68-2.92) | | 0.4 | |
| HLA-match | 10/10 | 1 |  | 1 | | |  | 1 | | |  | 1 | |  | |
|  | ≤ 9/10 | 1.36 (1.13-1.63) | 0.001 | 1.36 (1.15-1.62) | | | <0.001 | 1.30 (1.02-1.67) | | | 0.04 | 1.42 (1.12-1.80) | | 0.004 | |
| Stem cell source | Peripheral Blood Stem Cells | 1 |  | 1 | | |  | 1 | | |  | 1 | |  | |
|  | Bone Marrow | 0.91 (0.68-1.21) | 0.5 | 0.91 (0.69-1.19) | | | 0.5 | 1.13 (0.80-1.58) | | | 0.5 | 0.68 (0.44-1.05) | | 0.08 | |

Legend: HLA, human leukocyte antigen; Hazard ratios were calculated in multivariable Cox regression models stratified by registry (CIBMTR or EBMT), and adjusted for patient age, donor age, diagnosis, disease risk index, Karnofsky performance status, conditioning intensity, T-cell depletion, sex match, CMV match, HLA-match, and stem cell source.
