# Supplementary material for: Haplotype Motif-Based Models for KIR-Genotype Informed Selection of Hematopoietic Cell Donors Fail to Predict Outcome of Patients With Myelodysplastic Syndromes or Secondary Acute Myeloid Leukemia
Source: Front Immunol. 2021 Jan 19;11:584520. doi: 10.3389/fimmu.2020.584520 (PMC7851088; doi:10.3389/fimmu.2020.584520)
Supplement: Supplementary file 1 [file DataSheet_1.zip › Table S2A.DOC]

## Table S2A. Impact of *KIR* haplotype motif-based Models in Subgroups of Patients

| **Classifier** | **N** | **%** | **Relapse Incidence** | | **Event-free Survival** | |
| --- | --- | --- | --- | --- | --- | --- |
|  |  |  | HR (95%-CI) | P | HR (95%-CI) | P |
| **Patients with Secondary AML only** | | | | | | |
| *Cen A/A* | 233 | (49) | 1 |  | 1 |  |
| *Cen A/B* | 195 | (41) | 1.07 (0.75-1.52) | .7 | 0.93 (0.71-1.21) | .6 |
| *Cen B/B* | 45 | (10) | 0.73 (0.40-1.33) | .3 | 0.78 (0.50-1.21) | .3 |
|  |  |  |  |  |  |  |
| *Tel A/A* | 277 | (59) | 1 |  | 1 |  |
| *Tel A/B* | 177 | (37) | 0.98 (0.68-1.40) | .9 | 1.12 (0.86-1.46) | .4 |
| *Tel B/B* | 19 | (4) | 0.92 (0.41-2.06) | .8 | 0.86 (0.46-1.62) | .6 |
|  |  |  |  |  |  |  |
| “neutral” KIR-score (*B*≤1) | 332 | (70) | 1 |  | 1 |  |
| “better” KIR-score (any other) | 96 | (20) | 0.86 (0.57-1.32) | .5 | 0.90 (0.66-1.24) | .5 |
| “best” KIR-score (*Cen-B/B* ) | 45 | (10) | 0.68 (0.38-1.23) | .2 | 0.79 (0.51-1.21) | .3 |
| **Patients with MDS only** | | | | | | |
| *Cen A/A* | 565 | (46) | 1 |  | 1 |  |
| *Cen A/B* | 540 | (44) | 1.23 (0.96-1.58) | .10 | 1.13 (0.96-1.33) | .15 |
| *Cen B/B* | 126 | (10) | 1.48 (1.03-2.11) | .03 | 0.90 (0.68-1.19) | .4 |
|  |  |  |  |  |  |  |
| *Tel A/A* | 729 | (59) | 1 |  | 1 |  |
| *Tel A/B* | 442 | (36) | 1.13 (0.89-1.44) | .3 | 0.98 (0.83-1.16) | .8 |
| *Tel B/B* | 60 | (5) | 1.52 (0.92-2.49) | .10 | 1.16 (0.81-1.67) | .4 |
|  |  |  |  |  |  |  |
| “neutral” KIR-score (*B*≤1) | 841 | (68) | 1 |  | 1 |  |
| “better” KIR-score (any other) | 264 | (21) | 1.27 (0.96-1.67) | .10 | 1.06 (0.87-1.28) | .6 |
| “best” KIR-score (*Cen-B/B* ) | 126 | (10) | 1.42 (1.00-2.00) | .048 | 0.86 (0.65-1.13) | .3 |
| **Patients who received myeloablative conditioning** | | | | | | |
| *Cen A/A* | 248 | (48) | 1 |  | 1 |  |
| *Cen A/B* | 226 | (43) | 1.54 (1.07-2.21) | .02 | 1.32 (1.02-1.69) | .03 |
| *Cen B/B* | 48 | (9) | 1.35 (0.75-2.42) | .3 | 0.98 (0.62-1.54) | .9 |
|  |  |  |  |  |  |  |
| *Tel A/A* | 315 | (60) | 1 |  | 1 |  |
| *Tel A/B* | 189 | (36) | 1.05 (0.74-1.49) | .8 | 0.99 (0.77-1.27) | .9 |
| *Tel B/B* | 18 | (3) | 0.68 (0.21-2.22) | .5 | 1.02 (0.53-1.96) | 1,0 |
|  |  |  |  |  |  |  |
| “neutral” KIR-score (*B*≤1) | 361 | (69) | 1 |  | 1 |  |
| “better” KIR-score (any other) | 113 | (22) | 1.34 (0.91-1.99) | .14 | 1.12 (0.84-1.49) | .4 |
| “best” KIR-score (*Cen-B/B* ) | 48 | (9) | 1.17 (0.67-2.06) | .6 | 0.88 (0.57-1.37) | .6 |

Table S2A continued.

| **Classifier** | **N** | **%** | **Relapse Incidence** | | **Event-free Survival** | |
| --- | --- | --- | --- | --- | --- | --- |
|  |  |  | HR (95%-CI) | P | HR (95%-CI) | P |
| **Patients who received non-myeloablative or reduced-intensity conditioning** | | | | | | |
| *Cen A/A* | 529 | (46) | 1 |  | 1 |  |
| *Cen A/B* | 489 | (43) | 1.00 (0.78-1.29) | 1.0 | 0.92 (0.77-1.09) | .3 |
| *Cen B/B* | 122 | (11) | 1.15 (0.80-1.66) | .4 | 0.80 (0.61-1.07) | .13 |
|  |  |  |  |  |  |  |
| *Tel A/A* | 669 | (59) | 1 |  | 1 |  |
| *Tel A/B* | 413 | (36) | 1.07 (0.84-1.37) | .6 | 1.01 (0.85-1.20) | .9 |
| *Tel B/B* | 58 | (5) | 1.55 (0.97-2.46) | .06 | 1.08 (0.75-1.55) | .7 |
|  |  |  |  |  |  |  |
| “neutral” KIR-score (*B*≤1) | 782 | (69) | 1 |  | 1 |  |
| “better” KIR-score (any other) | 236 | (21) | 1.03 (0.77-1.38) | .8 | 0.94 (0.77-1.16) | .6 |
| “best” KIR-score (*Cen-B/B* ) | 122 | (11) | 1.16 (0.82-1.65) | .4 | 0.83 (0.63-1.09) | .2 |

Legend: Haplotype motifs and KIR scores were classed according to Cooley *et al*(9) (10). Hazard ratios were calculated in multivariable Cox regression models stratified by registry (CIBMTR or EBMT), and adjusted for patient age, donor age, diagnosis, disease risk index, Karnofsky performance status, conditioning intensity, T-cell depletion, sex match, CMV match, HLA-match, and stem cell source.
