# Supplementary material for: Haplotype Motif-Based Models for KIR-Genotype Informed Selection of Hematopoietic Cell Donors Fail to Predict Outcome of Patients With Myelodysplastic Syndromes or Secondary Acute Myeloid Leukemia
Source: Front Immunol. 2021 Jan 19;11:584520. doi: 10.3389/fimmu.2020.584520 (PMC7851088; doi:10.3389/fimmu.2020.584520)
Supplement: Supplementary file 1 [file DataSheet_1.zip › Table S2B.DOC]

## Supplemental Table 2B. Impact of *KIR* haplotype motif-based Models in Subgroups of C1-positive Patients

| **Classifier** | **N** | **%** | **Relapse Incidence** | | **Event-free Survival** | |
| --- | --- | --- | --- | --- | --- | --- |
|  |  |  | HR (95%-CI) | P | HR (95%-CI) | P |
| **C1+ Patients with Secondary AML only** | | | | | | |
| ***Cen A/A*** | 190 | (49) | 1 |  | 1 |  |
| ***Cen A/B*** | 160 | (41) | 1.07 (0.73-1.58) | 0.7 | 0.89 (0.66-1.19) | 0.4 |
| ***Cen B/B*** | 41 | (11) | 0.69 (0.36-1.32) | 0.3 | 0.75 (0.47-1.20) | 0.2 |
|  |  |  |  |  |  |  |
| ***Tel A/A*** | 232 | (59) | 1 |  | 1 |  |
| ***Tel A/B*** | 143 | (37) | 0.95 (0.64-1.42) | 0.8 | 1.01 (0.76-1.36) | 0.9 |
| ***Tel B/B*** | 16 | (4) | 0.88 (0.36-2.16) | 0.8 | 0.77 (0.38-1.57) | 0.5 |
|  |  |  |  |  |  |  |
| **“neutral” KIR-score (*B*≤1)** | 275 | (70) | 1 |  | 1 |  |
| **“better” KIR-score (any other)** | 75 | (19) | 0.81 (0.50-1.30) | 0.4 | 0.77 (0.54-1.11) | 0.2 |
| **“best” KIR-score (*Cen-B/B*)** | 41 | (11) | 0.64 (0.34-1.19) | 0.2 | 0.75 (0.48-1.18) | 0.2 |
| **C1+ Patients with MDS only** | | | | | | |
| ***Cen A/A*** | 470 | (45) | 1 |  | 1 |  |
| ***Cen A/B*** | 469 | (45) | 1.22 (0.93-1.60) | 0.15 | 1.13 (0.95-1.36) | 0.2 |
| ***Cen B/B*** | 107 | (10) | 1.71 (1.17-2.50) | 0.01 | 1.03 (0.77-1.39) | 0.8 |
|  |  |  |  |  |  |  |
| ***Tel A/A*** | 613 | (59) | 1 |  | 1 |  |
| ***Tel A/B*** | 382 | (37) | 1.02 (0.78-1.32) | 0.9 | 0.94 (0.78-1.13) | 0.5 |
| ***Tel B/B*** | 51 | (5) | 1.53 (0.89-2.64) | 0.12 | 1.21 (0.82-1.80) | 0.3 |
|  |  |  |  |  |  |  |
| **“neutral” KIR-score (*B*≤1)** | 709 | (68) | 1 |  | 1 |  |
| **“better” KIR-score (any other)** | 230 | (22) | 1.17 (0.87-1.58) | 0.3 | 1.00 (0.81-1.24) | 1.0 |
| **“best” KIR-score (*Cen-B/B*)** | 107 | (10) | 1.61 (1.12-2.32) | 0.01 | 0.97 (0.73-1.30) | 0.9 |
| **C1+ Patients who received myeloablative conditioning** | | | | | | |
| ***Cen A/A*** | 203 | (47) | 1 |  | 1 |  |
| ***Cen A/B*** | 188 | (43) | 1.45 (0.97-2.18) | 0.07 | 1.30 (0.98-1.72) | 0.07 |
| ***Cen B/B*** | 42 | (10) | 1.37 (0.72-2.58) | 0.3 | 1.01 (0.63-1.64) | 1.0 |
|  |  |  |  |  |  |  |
| ***Tel A/A*** | 262 | (61) | 1 |  | 1 |  |
| ***Tel A/B*** | 155 | (36) | 0.98 (0.66-1.45) | 0.9 | 0.93 (0.70-1.23) | 0.6 |
| ***Tel B/B*** | 16 | (4) | 1.00 (0.30-3.33) | 1.0 | 1.31 (0.65-2.64) | 0.5 |
|  |  |  |  |  |  |  |
| **“neutral” KIR-score (*B*≤1)** | 299 | (69) | 1 |  | 1 |  |
| **“better” KIR-score (any other)** | 92 | (21) | 1.29 (0.83-2.01) | 0.3 | 1.10 (0.80-1.52) | 0.6 |
| **“best” KIR-score (*Cen-B/B*)** | 42 | (10) | 1.21 (0.66-2.24) | 0.5 | 0.92 (0.58-1.46) | 0.7 |
| **C1+ Patients who received non-myeloablative or reduced-intensity conditioning** | | | | | | |
| ***Cen A/A*** | 439 | (45) | 1 |  | 1 |  |
| ***Cen A/B*** | 428 | (44) | 1.06 (0.81-1.38) | 0.7 | 0.93 (0.77-1.12) | 0.5 |
| ***Cen B/B*** | 105 | (11) | 1.35 (0.92-1.98) | 0.13 | 0.90 (0.67-1.21) | 0.5 |
|  |  |  |  |  |  |  |
| ***Tel A/A*** | 565 | (58) | 1 |  | 1 |  |
| ***Tel A/B*** | 358 | (37) | 0.95 (0.73-1.24) | 0.7 | 0.95 (0.79-1.15) | 0.6 |
| ***Tel B/B*** | 49 | (5) | 1.41 (0.85-2.33) | 0.2 | 1.04 (0.70-1.54) | 0.8 |
|  |  |  |  |  |  |  |
| **“neutral” KIR-score (*B*≤1)** | 661 | (68) | 1 |  | 1 |  |
| **“better” KIR-score (any other)** | 206 | (21) | 0.92 (0.67-1.26) | 0.6 | 0.87 (0.69-1.08) | 0.2 |
| **“best” KIR-score (*Cen-B/B*)** | 105 | (11) | 1.28 (0.89-1.85) | 0.2 | 0.90 (0.68-1.20) | 0.5 |

Supplemental Table 2B continued.

| **Classifier** | **N** | **%** | **Relapse Incidence** | | **Event-free Survival** | |
| --- | --- | --- | --- | --- | --- | --- |
|  |  |  | HR (95%-CI) | P | HR (95%-CI) | P |

| **Effects of single KIR genes which compose haplotype B motifs** | | | | | | |
| --- | --- | --- | --- | --- | --- | --- |
| ***KIR A/A*** | 429 | (30) | 1 |  | 1 |  |
| ***KIR B*/x with *2DS1*** | 558 | (39) | 1.10 (0.86-1.42) | 0.4 | 1.00 (0.84-1.19) | 1.0 |
| ***KIR B*/x with *2DS2*** | 776 | (54) | 1.16 (0.92-1.46) | 0.2 | 1.04 (0.88-1.22) | 0.7 |
| ***KIR B*/x with *2DS3*** | 428 | (30) | 1.13 (0.86-1.47) | 0.4 | 0.99 (0.82-1.19) | 0.9 |
| ***KIR B*/x with *2DS5*** | 410 | (29) | 1.04 (0.79-1.36) | 0.8 | 0.96 (0.79-1.16) | 0.7 |
| ***KIR B*/x with *2DL2*** | 765 | (53) | 1.14 (0.90-1.44) | 0.3 | 1.03 (0.87-1.21) | 0.7 |
| ***KIR B*/x with *2DL5*** | 714 | (50) | 1.07 (0.85-1.37) | 0.6 | 0.98 (0.83-1.16) | 0.8 |
| ***KIR B*/x with *3DS1*** | 557 | (39) | 1.09 (0.85-1.40) | 0.5 | 0.99 (0.83-1.18) | 0.9 |
